# Editorial: Targeting the PD-1/PD-L1 cancer immune evasion axis: Challenges and emerging strategies, Volume II

**DOI:** 10.3389/fphar.2022.1106129

**Published:** 2022-12-16

**Authors:** Xiujing He, Jie Xu, Huan Meng, Hubing Shi

**Affiliations:** ^1^ Laboratory of Integrative Medicine, Clinical Research Center for Breast, State Key Laboratory of Biotherapy, West China Hospital, Sichuan University and Collaborative Innovation Center, Chengdu, Sichuan, China; ^2^ Shanghai Key Laboratory of Medical Epigenetics, Institutes of Biomedical Sciences, Zhongshan-Xuhui Hospital, Fudan University, Shanghai, China; ^3^ CAS Key Laboratory for Biomedical Effects of Nanomaterials and Nanosafety, CAS Center for Excellence in Nanoscience National Center for Nanoscience and Technology, Beijing, China

**Keywords:** immunotherapy, biomarker, combination therapy, therapeutic toxicity, anti-cancer immunity

The onset of immune checkpoint blockade therapy (ICBT) over the last decade has dramatically revolutionized the treatment paradigm in cancer therapy. Immune checkpoint inhibitors (ICIs), especially monoclonal antibodies designed to block the programmed death 1/programmed death-ligand 1 (PD-1/PD-L1) axis, have scored impressive successes in malignant tumors and continue to show exceptional promise in ongoing clinical trials. However, the widespread utilization of ICIs is greatly hindered by their low response rates, severe adverse effects, innate and acquired drug resistance, etc. Increasing efforts are being made to overcome these issues and improve the clinical efficacy of ICBT. The aim of this Research Topic is to highlight novel and significant discoveries in the field of cancer immunotherapy, extending our understanding of the mechanisms of ICI-induced anti-cancer immunity, thereby improving therapeutic responses of ICBT and reducing the propensity for adverse events.

Considering the pitfalls of the existing antibody drugs targeting immune checkpoint molecules, small molecule inhibitors are desirable surrogates owing to their special properties, such as excellent cell permeability, high stability, cost-effectiveness, and low adverse events. Chen X. et al. regard tyrosine kinase inhibitor Bafetinib as a promising drug targeting PD-L1 that remarkably inhibits PD-L1 protein expression by suppressing the transcription of c-Myc. This study underscores the prospect of c-Myc as a druggable target and provides a new idea for tumor immunotherapy. The ongoing intensive work to mine small molecule inhibitors for clinical immunotherapy holds great promise for maximizing the patient benefit from these innovative therapies.

Accumulating evidence indicates that conventional therapeutics could synergize with ICI therapy, by facilitating cancer antigen release and presentation, reshaping the tumor microenvironment, or enhancing effector activity. Therefore, intense interest exists in developing combination therapies to boost the anti-tumor effects of ICIs. Currently, numerous therapeutic strategies that combine ICIs with standard treatment modalities, such as radiotherapy, chemotherapy, targeted therapy, and antiangiogenic therapy, are being evaluated in clinical settings. Ouyang et al. provide a current landscape of clinical trials of ICIs for patients with hepatocellular carcinoma, including monotherapy or combination therapy. Numerous positive findings from these clinical trials support the rationale of combining ICIs with other therapeutics in cancer therapy. Zheng et al. also present a comprehensive overview of recent advances in combinatorial strategies with PD-1/PD-L1 blockade for breast cancer therapy. As expected, anti-PD-1/PD-L1 therapy combined with chemotherapy, anti-HER2 therapy, and targeted therapy showed synergistic anti-tumor efficacies in the treatment of breast cancer. Nevertheless, several combination options only markedly improved the outcomes of specific subgroups of breast cancer, indicating the importance of patient selection for the optimal benefit of combination therapies.

Several emerging combination strategies have been proposed to circumvent the pitfalls of immunotherapy and expand the patient population benefiting from immunotherapy. Tryptophan metabolism enzymes indoleamine 2,3-dioxygenase 1 (IDO1) and tryptophan 2,3-dioxygenase 2 (TDO2) are partly responsible for ICIs resistance, thus the combination of IDO1/TDO2 inhibitors with ICI is a promising strategy to activate potent anti-tumor immunity. Zhang et al. found that sodium tanshinone IIA sulfonate (STS), a dual inhibitor of IDO1/TDO2, could decrease kynurenine synthesis, resulting in the lower kynurenine level in the plasma of murine model of colorectal cancer (CRC). Further analysis demonstrated that STS could suppress tumor growth and effectively boost tumor immunotherapy by decreasing the number of FOXP3^+^ T-cell and increasing the proportion of CD8^+^ T-cell in tumors. The potential of combined STS and immunotherapy for the synergistic treatment of CRC remains to be investigated. Yan et al., in their case report, found that the patient with ROS1 fusion-positive non-small cell lung cancer achieved a durable response to ICI plus chemotherapy, indicating that immuno-chemotherapy is a feasible option for ROS1-altered lung cancer. Additionally, they emphasized the importance of the sequence of combination treatments and proposed that TKI followed by ICI is a safer option. Yu et al. focus on the immune landscape during MAPK-targeted therapy to explore the mechanism of cross-resistance to ICIs driven by MAPK-targeted therapy. Consistent with previous reports, they found that BRAFi/MEKi acquired resistance was associated with reduced T-cell infiltration, implying that anti-tumor activity was suppressed. Transcriptomic dissection further suggested that deficiency of MHC-I antigen presentation on tumor cells might mediate drug-induced immunosuppression. Besides cross-resistance, aggravated therapeutic toxicity induced by combination therapy is another formidable challenge.

The establishment of predictive and prognostic biomarkers for monotherapy or combination therapy of checkpoint immunotherapy is essential to improve patient selection and avoid therapeutic toxicity. Compared with tissue-based biomarkers, such as PD-1/PD-L1 status, tumor mutational burden, and tumor-infiltrating lymphocytes, numerous blood parameters have increasingly captured the attention of researchers due to their non-invasive, cost-effective, and easy-to-use nature, which are prognostic of outcomes in several cancer types. Chen L. et al. assess the prognostic value of the gastric immune prognostic index (GIPI) in gastric cancer patients receiving anti-PD-1/PD-L1 therapy, which is available for liquid biopsy and reflects systemic inflammatory status. The result showed that high GIPI confers a stronger benefit to patients with gastric cancer.

As mentioned above, ICIs alone or in combination with other cancer therapeutics have exhibited momentous clinical benefits. It is noteworthy that the appropriate timing and sequence of combined treatments are important to attain optimal outcomes of combination therapy. These original research and reviews in this Research Topic help to buttress the potential of immunotherapy and provide important insights for anti-tumor immunity. Several potential effective combination strategies for cancer immunotherapy have been proposed, and further validation in clinical trials is needed. Moreover, molecular mechanisms underlying synergistic or antagonistic effects of combination therapy need further investigation, which will help deepen the understanding of anti-tumor responses and promote the development of promising therapeutic strategies.

